# Importance of the Brain Angiotensin System in Parkinson's Disease

**DOI:** 10.1155/2012/860923

**Published:** 2012-11-07

**Authors:** John W. Wright, Joseph W. Harding

**Affiliations:** Departments of Psychology, Veterinary and Comparative Anatomy, Pharmacology, and Physiology and Programs in Neuroscience and Biotechnology, Washington State University, P.O. Box 644820, Pullman, WA 99164-4820, USA

## Abstract

Parkinson's disease (PD) has become a major health problem affecting 1.5% of the world's population over 65 years of age. As life expectancy has increased so has the occurrence of PD. The primary direct consequence of this disease is the loss of dopaminergic (DA) neurons in the substantia nigra and striatum. As the intensity of motor dysfunction increases, the symptomatic triad of bradykinesia, tremors-at-rest, and rigidity occur. Progressive neurodegeneration may also impact non-DA neurotransmitter systems including cholinergic, noradrenergic, and serotonergic, often leading to the development of depression, sleep disturbances, dementia, and autonomic nervous system failure. L-DOPA is the most efficacious oral delivery treatment for controlling motor symptoms; however, this approach is ineffective regarding nonmotor symptoms. New treatment strategies are needed designed to provide neuroprotection and encourage neurogenesis and synaptogenesis to slow or reverse this disease process. The hepatocyte growth factor (HGF)/c-Met receptor system is a member of the growth factor family and has been shown to protect against degeneration of DA neurons in animal models. Recently, small angiotensin-based blood-brain barrier penetrant mimetics have been developed that activate this HGF/c-Met system. These compounds may offer a new and novel approach to the treatment of Parkinson's disease.

## 1. Introduction

Parkinson's disease (PD) was first described by James Parkinson in 1867 and now affects approximately 1.5% of the world's population over 65 years of age [1]. This disease is characterized by a progressive loss of dopaminergic (DA) neurons in the substantia nigra pars compacta. The striatum is the primary projection field of these substantia nigra neurons, thus the loss of DA results in insufficient stimulation of dopaminergic *D*_1_ and *D*_2_ receptors throughout the striatum [2–4]. Decreased availability of DA triggers the symptomatic triad of bradykinesia, tremors-at-rest, and rigidity. The pathogenesis of PD is unclear with both genetic and environmental factors playing roles. There is evidence from animal models and PD patients that neuroinflammatory processes, triggered by reactive oxygen species, damage mitochondrial membrane permeability, enzymes, and mitochondrial genome resulting in DA cell death [5, 6]. Progressive neurodegeneration may also impact non-DA neurotransmitter systems such as cholinergic, noradrenergic, and serotonergic. This expanded neural damage adds nonmotor symptoms such as sleep disturbances, depression, dementia, and possibly autonomic nervous system failure. L-DOPA is efficacious at controlling motor symptoms in the majority of patients but is ineffective regarding nonmotor symptoms. Current treatment strategies to relieve these symptoms include DA replacement via levodopa (L-DOPA, the precursor of DA), DA receptor agonists, monoamine oxidase B inhibitors, and catechol-O-methyltransferase inhibitors (to protect the DA that is formed). As the disease progresses periods of decreased mobility, dyskinesia, and spontaneous involuntary movements complicate treatment [7]. These motor dysfunctions are currently treated with the DA receptor agonists, apomorphine and levodopa, and surgical techniques including pallidectomy and deep brain electrical stimulation [8–10]. Progressive neurodegeneration may also involve additional nondopaminergic neurotransmitter systems including noradrenergic, cholinergic, and serotonergic [11]. As a result, nonmotor symptoms may develop including depression, sleep disturbances, dementia, and autonomic nervous system failure [12, 13].

L-DOPA continues to be the most efficacious oral delivery treatment for the control of motor symptoms [14]. Unfortunately, L-DOPA is reasonably ineffective at combating nonmotor symptoms [12]. Thus, current research efforts are directed at controlling these additional symptoms, as well as the development of new strategies designed to offer neuroprotection and overall disease reversal benefits. Attaining the goal of slowing or reversing the rate of DA neuron loss may also result in the protection of non-DA neurotransmitter systems. 

This paper focuses on a new target for the treatment of this disease, specifically the brain renin-angiotensin system (RAS), and the recent discovery of its interaction with hepatocyte growth factor (HGF) and its tyrosine kinase c-Met receptor [15, 16]. The HGF/c-Met receptor system functions as a critical survival system for motor and sensory neurons and a subset of root ganglion neurons [17–19]. This relationship offers interesting possibilities with respect to neurotransmitter systems crosstalk, suggesting that small angiotensin-based agonists and antagonists can be designed to act at the HGF/c-Met complex in place of large protein ligands. The next sections provide summaries of the RAS and HGF systems, consideration of reports describing their interaction, and the involvement of the RAS and HGF systems in PD. We conclude by presenting support for the notion that angiotensin agonists may be useful in activating the HGF/c-Met receptor system in order to provide cerebroprotection and encourage synaptogenesis in Parkinson's disease patients.

## 2. Brain Angiotensins and the AT_1_, AT_2_, and AT_4_ Receptor Subtypes

**Figure 1:**
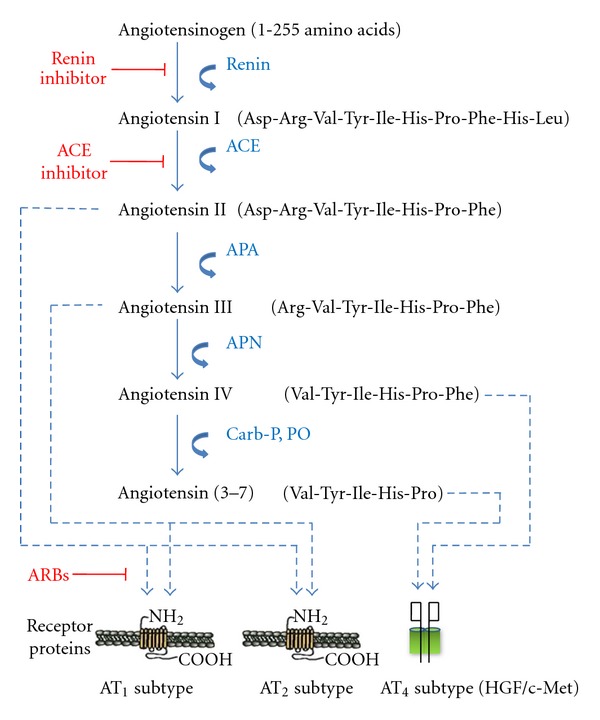
Description of the peptide structures and enzymes involved in the conversion of angiotensinogen to angiotensin I through shorter angiotensins. The biologically active forms include angiotensins II, III, IV, and angiotensin (3–7). The respective receptors where these angiotensins bind are indicated by arrows. The locations of action of angiotensin inhibitors are also indicated. Abbreviations: ACE = angiotensin converting enzyme; APA = aminopeptidase A; APN = aminopeptidase N; ARB = angiotensin receptor blocker; Carb-P = carboxy peptidase P; PO = propyl oligopeptidase.

The renin-angiotensin-aldosterone system is well known as a regulator of systemic blood pressure, body water balance, activation of sympathetic pathways, and control over vasopressin and oxytocin synthesis and release [20–22]. These functions are mediated, in part, by an independent brain RAS complete with the necessary components including angiotensinogen, renin, angiotensin converting enzyme (ACE), angiotensin ligands, and receptor proteins [23–26]. Following the discovery of this independent brain RAS separate from the peripheral system, three brain angiotensin receptor subtypes were identified. The first two, AT_1_ and AT_2_, are G-protein coupled and have been well described in previous review papers [15, 20, 22, 27] (Figure 1). Our laboratory discovered a third subtype, AT_4_, and its identity is currently a matter of controversy (see below).

The distribution of brain structures possessing AT_1_ receptor sites is reasonably consistent among the mammalian species examined using quantitative autoradiography and radioreceptor binding homogenate tissue preparations. These species include rat, mouse, hamster, dog, monkey, and human (reviewed in [28–30]). The AT_1_ subtype is localized in high densities within the anterior pituitary, area postrema, lateral geniculate body, inferior olivary nucleus, median eminence, nucleus of the solitary tract, the anterior ventral third ventricle region, paraventricular, preoptic and supraoptic nuclei of the hypothalamus, subfornical organ, and ventral tegmental area. This receptor subtype is represented in the following motor related brain structures: caudate putamen, cerebellum, striatum, and substantia nigra (Table 1).

The highest densities of the AT_2_ site are found in the amygdala, medial geniculate body, habenula, hypoglossal nucleus, inferior colliculus, inferior olivary nucleus, locus coeruleus, striatum, thalamus, and ventral tegmental area. This receptor subtype is present in the following motor related structures: caudate putamen, cerebellum, globus pallidus, and substantia nigra (Table 1). 

The AT_4_ receptor is distributed within a number of brain structures with notably high concentrations in the anterior pituitary, cerebral cortex, lateral geniculate body, habenula, hippocampus, inferior olivary nucleus, nucleus basalis of Meynert, periaqueductal gray, piriform cortex, superior colliculus, thalamus, and ventral tegmental area, and of particular interest caudate putamen, cerebellum, globus pallidus, nucleus accumbens, red nucleus, striatum, and substantia nigra (Table 1). Although the brain distribution of AngIV is not available, the locations of aminopeptidase A (AP-A, an aminopeptidase that converts the octapeptide AngII to the heptapeptide AngIII) and aminopeptidase N (AP-N, an aminopeptidase that converts AngIII to the hexapeptide AngIV) are suggestive given their likely co-localization with AngIV. Both AP-A and AP-N have been localized to the plasma membrane of pericytes suggesting that AngIV is found in the extracellular space surrounding microvessels in the brain [34, 35]. In support of this notion exogenous administration of AngIV has been shown to increase cerebral microcirculation [36–38]. Most relevant, Lanckmans and colleagues [39, 40] measured AngIV in the striatum using microdialysis coupled with a sensitive liquid chromatography mass spectrometry system. However, shortly following probe insertion the levels of AngIV often dropped below the detection limit of 50 pM. This was interpreted to suggest an intracellular presence for AngIV. This notion is supported by several reports indicating that within neurons AngII is converted to AngIV (80%), with smaller fractions of AngIII, Ang(1–7), and Ang(1–6) (reviewed in [41]).

Thus, of the three subtypes the AT_4_ receptor, colocalized with AngIV, is prominently represented in brain structures associated with motor functioning; however, to date the greatest attention has been devoted to the AT_1_ and AT_2_ receptor subtypes. Other functions associated with each angiotensin receptor subtype are presented in Table 2.

## 3. Brain Hepatocyte Growth Factor/c-Met 

Hepatocyte growth factor, also known as “scatter factor”, is a glycoprotein recognized as a potent mitogenic, morphogenic, and motogenic growth factor that acts via the type 1 tyrosine kinase receptor c-Met [42]. HGF was originally isolated from the liver and was shown to promote liver regeneration [43]. In 1991, Bottaro et al. [44] identified c-Met as a receptor for HGF. The c-Met receptor protein is made up of disulfide bond-linked alpha (45 kDA) and beta (145 kDa) subunits [45]. The alpha chain is extracellular while the beta chain is transmembrane. HGF dimerization precedes binding to the c-Met receptor which then undergoes phosphorylation. Once phosphorylated, the tyrosine residues of the beta subunit serve as docking sites for downstream signaling mediators including the extracellular signal-regulated kinase (ERK) and the phosphatidylinositol-3-kinase (P13K) pathway [46, 47]. This HGF/c-Met signaling is regulated by the activator, hepatocyte growth factor A (HGFA), and its inhibitor, HGFAI. HGFA is a protease that acts on the precursor protein and produces active HGF. In contrast, HGFAI blocks the activation of HGFA [48]. c-Met has been shown to play a role in multiple types of cancer (reviewed in [49, 50]), blunt neurodegenerative changes [51], facilitate long-term potentiation (LTP [52]), contribute to learning and memory consolidation [52–56], and may play a role in Alzheimer's disease [57, 58]. Also, inactivation of c-Met in the embryonic proliferative zones of mice results in an increase in parvalbumin-expressing cells in the dentate gyrus, a loss of these cells in the CA3 field, with an overall loss of calretinin-expressing cells throughout the hippocampus [59]. These results highlight the importance of c-Met with regard to appropriate hippocampal development. Lan et al. [60] have shown that HGF regulates proliferation and migration of dopaminergic progenitor cells isolated from fetal striatum. These cells were capable of differentiating into functioning neurons with the ability to release DA. Schwartz and colleagues [61] have reported that human embryonic stem cell-derived dopaminergic neurons increased expression of tyrosine hydroxylase (a DA neuron marker) when any one of several growth factors were added to the cell culture including HGF, stromal cell-derived factor-1*α*, and vascular endothelial growth factor. The authors concluded that these growth factors may be of potential use to induce DA cellular differentiation of pluripotent human stem cells.

There have been reports of elevated levels of cerebrospinal fluid HGF in PD patients as compared with normal controls [62, 63]. Along these lines, several researchers have suggested the use of HGF as a therapeutic agent for amyotrophic lateral sclerosis and neuroimmune diseases [19, 64], ischemia-stroke [53, 65], neurodegenerative diseases [66], and CNS neuron survival [67–69]. Recently, Koike and colleagues [70] utilized the 6-hydroxy dopamine (6-OHDA) rat model of PD to test the hypothesis that transfected human HGF injected into the striatum could protect DA neurons. 6-OHDA lesioned rats treated with lacZ plasmid lost more than 90% of their DA neurons. In contrast, 70% of the DA neurons survived in rats transfected with HGF. Thus, over expression of HGF protected DA neurons in these 6-OHDA lesioned rats. These results are important for two reasons: (1) a gene therapy approach designed to overexpress HGF may be efficacious when applied to PD patients and (2) these results indicate that a drug designed to facilitate HGF expression in PD patients may offer neuroprotection from ongoing DA neurodegeneration.

## 4. Interaction between Angiotensin IV and the HGF/c-Met System

Although the identity of the AT_4_ receptor remains controversial, this receptor protein has been partially sequenced as insulin-regulated aminopeptidase (IRAP [71, 72]). The distribution of brain IRAP mRNA and protein matches that of the AT_4_ receptor protein as indicated by [^125^I] AngIV-radioligand binding assay [71, 73]. IRAP is a member of type 2 transmembrane proteins of the gluzincin aminopeptidase family [74] which includes homologous aminopeptidases such as aminopeptidases A and N. IRAP is capable of cleaving the N-terminal amino acid from a number of peptides including met-enkephalin, dynorphin, oxytocin, arginine-vasopressin, lysine-bradykinin, neurokinin A, somatostatin, neuromedin B, and cholecystokinin-8 [75–77]. Thus, IRAP has been variously identified as oxytocinase, cystinyl aminopeptidase, placental leucine aminopeptidase, gp 160, or vp 165 depending on its independent cloning (reviewed in [78]). The key substrates acted upon by this enzyme are thought to be arginine vasopressin and oxytocin [72, 79]. IRAP consists of 1025 amino acid residues with a 110 amino acid N-terminal hydrophilic intracellular domain that includes two dileucine motifs. The hydrophobic transmembrane domain consists of 22 amino acids that continues with an 893 amino acid C-terminal extracellular domain associated with its catalytic site. The catalytic site is composed of a GAMEN motif and includes the HEXXH(X)_18_ Zn^2+^-binding motif [80–82]. 

Recently our laboratory has challenged the “AT_4_ receptor is IRAP” hypothesis. This challenge is based on our search for a molecular target with structural homology to angiotensin IV and physiological functions in agreement with those identified for the AngIV/AT_4_ system. We discovered a partial match with the antiangiogenic protein angiostatin and the related plasminogen family member HGF. The functions associated with the HGF/c-Met system overlap with those mediated by the AngIV/AT_4_ system including facilitated memory consolidation, augmented neurite outgrowth, hippocampal LTP and calcium signaling, dendritic arborization, facilitation of cerebral blood flow and cerebroprotection, seizure protection, and facilitated wound healing (Table 3; reviewed in [15, 16]). This led to the hypothesis that AngIV analogues may exert their activity via the HGF/c-Met system. In a recent investigation we reported that the AT_4_ receptor antagonist, Norleual-AngIV, inhibited HGF binding to c-Met and HGF-dependent signaling, proliferation, invasion, and scattering [83]. The mechanism of action regarding Norleual-AngIV's ability to act as a c-Met receptor antagonist is by inhibiting the dimerization of HGF which is a prerequisite to c-Met binding [84, 85]. These results strongly suggest that the biological effects of AngIV, and AngIV analogues, are mediated through the HGF/c-Met system. 

Several observations and research findings are relevant to the hypothesis that the AT_4_ receptor subtype is HGF/c-Met. (1) As mentioned earlier, heavy brain distributions of the AT_4_ receptor subtype are located in neocortex, piriform cortex, hippocampus, nucleus basalis of Meynert, amygdala, cerebellum, caudate putamen, globus pallidus, striatum, and substantia nigra, consistent with expectations concerning brain locations for a receptor acting as a mediator of cognitive and motor processing [28, 31, 118, 119]. Partial determination of brain c-Met receptor distributions generally agree with this pattern [120, 121]. (2) The AT_4_ receptor subtype's ability to facilitate LTP, separate from NMDA-dependent LTP, suggests a nonglutamatergic signaling pathway [93]. (3) The finding that facilitation of the AT_4_ receptor subtype results in increased internalization of calcium via at least three different calcium channels suggests a rapid and salient cell signaling event [93] and agrees with the observation that HGF-induced responses also depend upon the internalization of calcium [98]. (4) Conversion of AngII to AngIV appears to be necessary for AngII-induced DA release in the striatum [122], and acetylcholine release in the hippocampus [123]. (5) The coupling of increased neural intracellular calcium with matrix metalloproteinases released into the extracellular space suggests a neural plasticity function [124, 125]. (6) Recent neural imaging work completed in our laboratory (see below [97]) indicates that Nle^1^-AngIV stimulates dendritic spine numbers and size in the hippocampus, as well as overall dendritic arborization, suggesting a plausible synaptogenesis mechanism to explain the ability of these molecules to enhance synaptic plasticity and connectivity among neurons. In agreement, HGF has been shown to increase dendritic arborization in hippocampal neurons in culture [98]. 

Members of our research group have focused attention on understanding how AT_4_ receptor agonists and antagonists facilitate and interfere with, respectively, learning and memory. We determined that the metabolically resistant agonist Nle^1^-AngIV significantly facilitated LTP in the CA1 field of hippocampal slices [94], while both AngIV, and Nle^1^AngIV, enhanced LTP in the dentate gyrus *in vivo* [95]. Pretreatment with the AT_4_ receptor antagonist Divalinal-AngIV prior to tetanization significantly disrupted the maintenance phase of LTP. The Nle^1^-AngIV facilitation of LTP was shown to be dependent on increased intracellular calcium via L- and T-type voltage-dependent calcium channels [93]. The ability of these agonists to promote Ca^2+^ entry, particularly via L-type channels, suggested the potential mechanism of altered dendritic arborization [126, 127]. We next examined the ability of AT_4_ agonists to facilitate dendritic arborization in disassociated rat hippocampal neurons labeled with mRFP-bactin to visualize the cytoskeleton, including the spines. Quantitative analysis from neurons exposed to Nle^1^-AngIV for 5 days indicated an increased number of dendritic spines per dendrite, accompanied by an expansion in dendritic arborization [97]. The above observations support the hypothesis that the primary mechanism underlying memory facilitation by AngIV and its analogues may be the ability to enhance synaptic communication and neural activity.

These Nle^1^-AngIV-induced increases in dendritic arborization are consistent with the hypothesis that AT_4_ receptor ligands alter HGF docking at the c-Met receptor. There are several reports indicating that HGF and c-Met are neuronally expressed in several brain structures including the neocortex and hippocampus [120] and appear in high densities at excitatory synapses within the hippocampus [121]. Activation of the c-Met receptor by HGF promotes neurite outgrowth [128] and dendritic branching by cortical neurons in sliced cultures [99]. The complexity of the dendritic branching could be attenuated with anti-HGF antibodies [99]. Recently, Tyndall and colleagues [98] reported that HGF increased the size and complexity of dendritic arborization in dissociated hippocampal neurons in culture. This facilitation could be blocked by pretreatment with the NMDA receptor antagonist, DL-2-amino-5-phosphonopentanoic acid (APV). It was further determined that this HGF effect is dependent on elevations in intracellular calcium and accompanying increases in autophosphorylation of CaMKII. These results suggest that Ca^2+^-dependent processing underlies HGF's ability to increase dendritic arborization and are consistent with our findings indicating increased hippocampal neuronal intracellular calcium with Nle^1^-AngIV treatment and facilitated hippocampal dendritic arborization. Pretreatment of cultured hippocampal neurons with an AT_4_ receptor antagonist inhibited this Nle^1^-AngIV-induced arborization. Recently our laboratory has used a tritiated small molecule HGF analogue to further identify the locations of brain HGF/c-Met receptors [129]. Reasonably high concentrations of HGF/c-Met were measured in the prefrontal cortex, hippocampus, cerebellum, thalamus, hypothalamus, striatum, and lower brain stem structures.

## 5. A Link between the Brain Angiotensin System and Parkinson's Disease

The potential relationship between the brain RAS and PD was initially suggested by Allen and colleagues [130]. These investigators measured decreased angiotensin receptor binding in the substantia nigra and striatum in post mortem brains of PD patients. A number of studies support an important role for ACE in this disease. ACE is present in the nigra-striatal pathway and basal ganglia structures [131–133]. Parkinson's disease patients treated with the ACE inhibitor perindopril revealed improved motor responses to the DA precursor 3,4-dihydroxy-L-phenylalanine [134]. Relative to this treatment with perindopril, elevated striatal DA levels have been measured in mice [135]. In addition, ACE has been shown to metabolize bradykinin and thus modulate inflammation [136], a contributing factor in PD. Activation of the AT_1_ receptor subtype by AngII promotes nicotinamide adenine dinucleotide phosphate (NADPH)-dependent oxidases, a significant source of reactive oxygen species [137, 138]. Treatment with ACE inhibitors has been shown to offer protection against the loss of DA neurons in 1-methyl-4-phenyl-1,2,3,6-tetrahydropyridine (MPTP) animal models [139, 140], as well as the 6-OHDA rat model [141]. The likely mechanism underlying this ACE inhibitor-induced protection is a reduction in the synthesis of AngII acting at the AT_1_ receptor subtype (reviewed in [142]). It is known that AngII binding at the AT_1_ subtype activates the NADPH oxidase complex, thus providing a major source of reactive oxygen species [143, 144]. Further, activation of the AT_1_ receptor results in the stimulation of the NF-*κ*B signal transduction pathway facilitating the synthesis of chemokine, cytokines, and adhesion molecules, all important in the migration of inflammatory cells into regions of tissue injury [145]. 

Given the above reports, it follows that if AngII activation of the AT_1_ receptor subtype results in facilitation of the NADPH oxidase complex, and thus formation of free radicals, then blockade of the AT_1_ receptor should serve a protective function. This appears to be the case. Treatment with AT_1_ receptor antagonists, known as angiotensin receptor blockers (ARBs), protects DA neurons in both 6-OHDA [33, 146–148] and MPTP animal models [144, 149, 150]. ARBs have been shown to reduce the formation of NADPH oxidase-derived reactive oxygen species following administration of 6-OHDA [33]. While the risk of developing PD is reduced with the use of calcium channel blockers to control hypertension, the influence of ACE inhibitors, *β*-blockers, and ARBs is not clear [151]. Ascherio and Tanner [152] have pointed out several shortcomings in the above study by Becker and colleagues and suggested that their analysis be redone to include a time frame of up to two years prior to the onset of PD symptoms. Of relevance to this issue, there is the occasional PD patient in which an ARB (Losartan) has been reported to exacerbate the motor dysfunctions [153]. While on Losartan, this patient experienced severe bradykinesia accompanied by frequent episodes of freezing. 

The AT_2_ receptor subtype is present in several fetal tissues including uterus, ovary, adrenal gland, heart, vascular endothelium, kidney, and brain (particularly neocortex and hippocampus) [20, 154–157]. As the animal matures, the expression of the AT_2_ receptor decreases. It appears that adult mammalian brain levels of this receptor in the striatum and substantia nigra are reasonably low [22, 158]. The AT_2_ receptor has been linked with cell proliferation, differentiation, and tissue regeneration [159–162]. The results from a study utilizing mesencephalic precursor cells indicated that AngII, acting at the AT_2_ receptor, facilitated differentiation of precursor cells into DA neurons [163]. Along these lines, activation of the AT_2_ receptor has been shown to inhibit NADPH oxidase activation [164]. However, Rodriguez-Pallares et al. [165] found that AngII treatment of the 6-OHDA lesioned rat increased DA cell death. This could be due to the much greater numbers of brain AT_1_ receptors, as compared with AT_2_ receptors, such that the beneficial effects of AT_2_ receptor activation were overwhelmed by AT_1_ activation. Finally, the expression of AT_2_ receptors in PD patients appears to be decreased in the caudate nucleus but is unchanged in the substantia nigra and putamen [166].

Recent studies using several animal models indicate that basal ganglia structures possess a local RAS that evidences increased activity during dopaminergic degeneration [167–169]. For example, reserpine-induced decreases in DA resulted in a significant increase in the expression of AT_1_ and AT_2_ receptors [170]. A similar pattern was seen with 6-OHDA-induced DA denervation, with a decrease in receptor expression when L-dopa was given. These results are important in that a clear interaction between the RAS and the DA system appears to be present in basal ganglia structures. Related to this, Rodriguez-Perez and colleagues [171] produced dopaminergic degeneration via intrastriatal 6-OHDA injection and noted a significant decrease in dopaminergic neurons in ovariectomized rats. This neuron loss was attenuated by treatment with the AT_1_ receptor antagonist candesartan, or estrogen replacement. Estrogen replacement also resulted in a downregulation of AT_1_ receptors and NADPH complex in the substantia nigra, accompanied by an upregulation of the AT_2_ receptor subtype. These results indicate an important relationship among estrogen levels, brain DA receptors, and the RAS. An increase in the expression of AT_1_ receptors and decreased expression of AT_2_ receptors has been reported in aged rats [172]. This observation is of major importance given the potentially deleterious consequences of AT_1_ receptor activation on basal ganglia structures.

Recently the Rodriquez-Perez research group [173] reported that chronic hypoperfusion in rats resulted in a reduction in striatal DA levels, accompanied by a large decline in dopaminergic neurons and striatal terminals. This DA neuron loss was countered by orally administered candesartan. In addition, AT_1_ receptor expression was highest in the substantia nigra, while AT_2_ expression was lower in rats that experienced chronic hypoperfusion as compared with controls. Again these effects could be attenuated by candesartan. Taken together, these findings argue that inhibition of AT_1_ receptor activity should serve a neuroprotective role in PD.

The potential involvement of AngIV in Parkinson's disease has been initially investigated [110]. A genetic *in vitro* PD model was used consisting of the *α*-synuclein overexpression of the human neuroglioma H4 cell line. Results indicated a significant reduction in *α*-synuclein-induced toxicity with Losartan treatment combined with the AT_2_ receptor antagonist PD123319, in the presence of AngII. Under these same conditions, AngIV was only moderately effective. However, these researchers did not use a metabolically stable AngIV analogue, nor did they confirm effects with an AT_4_ receptor antagonist in combination with AngII or AngIV.

Overall, experimental work suggests that treatment with an ARB may offer some protection against the risk of developing PD. However, much additional work must be completed to better understand the relationship among brain angiotensin receptors, ligands, inflammation, and reactive oxygen species as related to PD.

## 6. Relationship among Angiotensins, HGF, and Parkinson's Disease

Aging is one of the major risk factors predisposing individuals to neurodegenerative diseases [174–177]. The neurodegeneration accompanying aging is dependent in part upon oxidative stress, neuroinflammation, and microglial NADPH oxidase activity. Each is of significant importance regarding DA neuron loss [178, 179]. Activation of AT_1_ receptors by AngII has been shown to facilitate DA neuron degeneration by activating microglial NADPH oxidase [147]. The activation of AT_1_ receptors by AngII failed to cause DA neuron degeneration when microglial cells were absent [180]. Of related importance, Zawada and colleagues [181] recently reported that nigral dopaminergic neurons respond to neurotoxicity-induced superoxide in two waves. First, a spike in mitochondrial hydrogen peroxide was measured three hours following treatment with an MPTP metabolite (MPP+). Second, by twenty-four hours following treatment, hydrogen peroxide levels were further elevated. Treatment with Losartan suppressed this nigral superoxide production suggesting a potentially important role for ARBs in the treatment of PD. Further, AngII binding at the AT_1_ receptor increased DA neuron degeneration initiated by subthreshold doses of DA neurotoxins by stimulating intraneuronal levels of reactive oxygen species (ROS) and neuroinflammation by activation of microglial NADPH oxidase [37, 144, 182–184].

From the above observations, it follows that AT_1_ receptor blockade should have a neuroprotective effect on DA neurons in PD patients as demonstrated in animal models [149]. Less obvious is the likelihood that AT_1_ receptor blockade results in accumulating levels of AngII which is converted to AngIII and then to AngIV. This conversion cascade has been shown to occur intracellularly [41]. In fact, this conversion of AngII appears to be necessary for DA release to occur in the striatum [122]. Thus, an intriguing alternative explanation of these AT_1_ receptor antagonist results is that the increased endogenous levels of AngIV facilitate activation of the HGF/c-Met receptor system and neuroprotection of DA neurons. In this way, AngIV may act in combination with AT_1_ receptor blockade to protect DA neurons. Our laboratory has offered evidence that AngIV, and AngIV analogues, are capable of acting to facilitate HGF/c-Met activity [97]. Support for this claim is presented in several recent reports. First we found that the action of AT_4_ receptor antagonists depends on inhibiting the HGF/c-Met receptor system by binding to and blocking HGF dimerization [83, 84]. In contrast, AT_4_ receptor agonists facilitate cognitive processing and synaptogenesis by acting as mimics of the dimerization domain of HGF (hinge region) [85]. This work has culminated in the synthesis of a small molecule AT_4_ receptor agonist capable of penetrating the blood-brain barrier and facilitating cognitive processing presumably by increasing synaptogenesis. This small molecule (MM-201) has a Kd for HGF *≈* 13 picomolar [129]. This AngIV-HGF/c-Met interaction could explain earlier reports indicating that activation of the AT_4_ receptor facilitates cerebral blood flow and neuroprotection [36, 38, 103]. 

In agreement with the above findings, HGF has been shown to positively impact ischemic-induced injuries such as cardiac [185] and hind limb ischemia [104, 105]. HGF has also been shown to eliminate hippocampal neuronal cell loss in transient global cerebral ischemic gerbils [65], and transient focal ischemic rats [106]. Date and colleagues [54] have reported HGF-induced improvements in escape latencies by microsphere embolism-cerebral ischemic rats using a circular water maze task. These authors measured reduced damage to cerebral endothelial cells in ischemic animals treated with HGF. Shimamura et al. [51] have recently shown that over-expression of HGF following permanent middle cerebral artery occlusion resulted in significant recovery of performance in the Morris water maze and passive avoidance conditioning tasks. Treatment with HGF was also found to increase the number of arteries in the neocortex some 50 days following the onset of ischemia. 

In sum, these results suggest a role for the HGF/c-Met receptor system in cerebroprotection and are consistent with the notion that AngIV increases blood flow by an NO-dependent mechanism [37]. In support of this hypothesis, a report by Faure et al. [113] indicated that increasing doses of AngIV via the internal carotid artery significantly decreased mortality and cerebral infarct size in rats twenty-four hours following embolic stroke due to the intracarotid injection of calibrated microspheres. Pretreatment with the AT_4_ receptor antagonist Divalinal-AngIV, or N*ω*-nitro-L-arginine methyl ester (L-NAME), abolished this protective effect. Sequential cerebral autoradiography indicated that AngIV caused the redistribution of blood flow to ischemic areas within a few minutes. Thus, AngIV may yield its cerebral protective effect against acute cerebral ischemia via an intracerebral-hemodynamic c-Met receptor-mediated NO-dependent mechanism. Should these relationships hold, then a metabolically stable blood-brain barrier penetrant small molecule compound that activates the HGF/c-Met system could prove highly efficacious in the treatment of PD.

## 7. Conclusion

Parkinson's disease is a major neurodegenerative disease that is increasing in patient numbers world wide as populations live longer. New treatment strategies are needed to slow or reverse this disease process. The HGF/c-Met receptor system may offer neuroprotection to dopaminergic neurotransmitter pathways. However, the direct use of HGF has at least two major problems: (1) HGF is a large heterodimeric protein that is very expensive to produce; (2) as a large protein, HGF does not penetrate the blood-brain barrier and thus cannot reach brain locations where neurodegeneration is occurring. We have discovered that the small peptide AngIV, and its analogues, cause HGF dimerization which is a prerequisite to binding and activation of the c-Met receptor [42]. HGF has been shown to be intimately involved in cell survival, proliferation, migration, and differentiation [186–188] and blunts neurodegenerative influences [51]. The availability of small molecule HGF mimetics represents a significant advantage over the use of large HGF analogues to accomplish the treatment goal of slowing or reversing PD-induced neurodegeneration. It remains to be seen whether long-term treatment of PD patient is possible and efficacious using small molecule HGF mimetics.

## Figures and Tables

**Table 1:** Predominant distributions of the three angiotensin receptor subtypes and the HGF/c-Met receptor identified in mammalian brains.

Subtype	AT_1_	AT_2_	AT_4_	c-Met
Structure				
Caudate putamen	+	++	++	
Cerebellum	+	+	++	++
Globus pallidus		++	++	
Nucleus accumbens			+	
Periaqueductal gray			++	
Red nucleus			+	
Striatum	++		++	++
Substantia nigra	++		+	
Ventral tegmental area	++	++	++	

Adapted from [15, 22, 30–33]; +: moderate levels of the receptor subtype; ++: high levels of the receptor subtype.

**Table 2:** Ligand activation of the AT_1_, AT_2_, and AT_4_ receptor subtypes influence the following functions.

AT_1_ receptor subtype	
Vasoconstriction	
Aldosterone release	
Vasopressin release	
Cardiac hypertrophy	
Fibrosis	
Proliferation	
Inflammation	
Platelet aggregation	
Oxidative stress	
Endothelial disruption	

AT_2_ receptor subtype	
Vasodilation	
Antifibrotic	
Antiproliferative	
Antihypertrophic	
Antithrombotic	

AT_4_ receptor subtype	
Dendritic arborization	
Changes in blood flow	
Memory facilitation	
Protection against seizures	
Facilitates wound healing	

**Table 3:** Summary of overlapping functions associated with the AngIV/AT_4_ receptor subtype and the HGF/c-Met receptor.

Function	AngIV/AT_4_ receptor subtype	HGF/c-Met receptor
Memory facilitation	[30, 86–92]	[51, 53, 54, 56, 69]
Hippocampal LTP, Ca^++^ signaling	[93–96]	[52]
Dendritic arborization	[97]	[98–102]
Cerebral blood flow	[36, 38, 103]	[51, 53, 54, 65, 104–106]
Seizure protection	[107–109]	[12]
Parkinson's disease	[110]	[60–63, 70]
Angiogenesis and PAI-1 expression	[22, 83, 111, 112]	[65, 113, 114]
Neurite outgrowth	[115]	[114, 116, 117]
